# Supplementary material for: Molecular Analyses of Clinical Isolates and Recombinant SARS-CoV-2 Carrying B.1 and B.1.617.2 Spike Mutations Suggest a Potential Role of Non-Spike Mutations in Infection Kinetics
Source: Viruses. 2022 Sep 12;14(9):2017. doi: 10.3390/v14092017 (PMC9506066; doi:10.3390/v14092017)
Supplement: Supplementary file 1 [file viruses-14-02017-s001.zip › viruses-1885221-supplementary/Supplemental Files-V2.pdf]

Molecular Analyses of Clinical Isolates and Recombinant SARS-CoV-2 Carrying B.1 and B.1.617.2 Spike Mutations Suggest a Potential Role of Non-Spike Mutations in Infection Kinetics

Andrei Veleanu [1,†], Maximilian A. Kelch [1,†], Chengjin Ye [2], Melanie Flohr [1], Alexander Wilhelm [1], Marek Widera [1], Luis Martinez-Sobrido [2], Sandra Ciesek [1,3,4] and Tuna Toptan [1,*]

Supplemental Tables and Figure

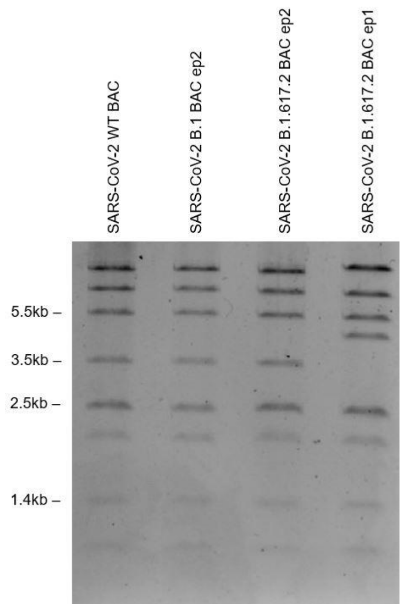

Figure S1: Restriction digestion analysis of different BAC clones. BAC DNA were digested with EcoRI-HF overnight and analyzed on an 0.7% TBE agarose-gel prestained with Midori Green Advance. Banding patterns following the second step of en passant mutagenesis (indicated with ep2, lane 2 and 3) are similar to WT-BAC (first lane). Plasmid containing the successful insertion of the kanamycin cassette and the duplication (indicated with ep1, lane 4) displays a shift of size in one fragment of 1.1 kb from a size of 3.5 kb to approximately 4.6 kb.

Video S1-S5 Live cell imaging of A549-AT cells infected with viruses given below, cells were kept under environmentally controlled conditions (37°C and 5% CO2), and images were acquired every 2 h for 44 h.

Video S1: $WT^{BAC\text{-}V}$

Video S2: $B.1^{BAC\text{-}V}$

Video S3: $B.1^{CI}$

Video S4: $B.1.617.2^{BAC\text{-}V}$

Video S5: $B.1.617.2^{CI}$

References

1. WHO. Molecular Assays to Diagnose COVID-19: Summary Table of Available Protocols. **2020**.
